# Loss of HES1 expression is associated with extracellular matrix remodeling and tumor immune suppression in *KRAS* mutant colon adenocarcinomas

**DOI:** 10.1038/s41598-023-42234-7

**Published:** 2023-09-25

**Authors:** Lei Wang, Wenchao Gu, Bingqing Zou, Matthew Kalady, Wei Xin, Lan Zhou

**Affiliations:** 1 Department of Pathology, Case Western Reserve University, Cleveland, OH USA; 2 Department of Diagnostic and Interventional Radiology, University of Tsukuba, Tsukuba, Ibaraki Japan; 3 Department of Colorectal Surgery, Cleveland Clinic, Cleveland, OH USA; 4 Department of Pathology, University Hospitals Cleveland Medical Center, Cleveland, OH USA; 5 Present Address: Department of Pathology, Fudan University Shanghai Cancer Center, Shanghai, China; 6 Present Address: Department of Pathology and Genomic Medicine, Houston Methodist Research Institute, Houston, TX USA; 7 Present Address: Division of Colon and Rectal Surgery, Ohio State University Wexner Medical Center and James Comprehensive Cancer Center, Columbus, OH USA; 8 Present Address: Department of Pathology, University of South Alabama Hospital, Mobile, AL USA

**Keywords:** Cancer, Computational biology and bioinformatics

## Abstract

The loss of HES1, a canonical Notch signaling target, may cooperate with *KRAS* mutations to remodel the extracellular matrix and to suppress the anti-tumor immune response. While HES1 expression is normal in benign hyperplastic polyps and normal colon tissue, HES1 expression is often lost in sessile serrated adenomas/polyps (SSAs/SSPs) and colorectal cancers (CRCs) such as those right-sided CRCs that commonly harbor *BRAF* or *KRAS* mutations. To develop a deeper understanding of interaction between KRAS and HES1 in colorectal carcinogenesis, we selected microsatellite stable (MSS) and *KRAS* mutant or *KRAS* wild type CRCs that show aberrant expression of HES1 by immunohistochemistry. By comparing the transcriptional landscapes of microsatellite stable (MSS) CRCs with or without nuclear HES1 expression, we investigated differentially expressed genes and activated pathways. We identified pathways and markers in the extracellular matrix and immune microenvironment that are associated with mutations in *KRAS*. We found that loss of HES1 expression positively correlated with matrix remodeling and epithelial-mesenchymal transition but negatively correlated with tumor cell proliferation. Furthermore, loss of HES1 expression in *KRAS* mutant CRCs correlates with a higher M2 macrophage polarization and activation of IL6 and IL10 immunosuppressive signature. Identifying these HES1-related markers may be useful for prognosis stratification and developing treatment for *KRAS*-mutant CRCs.

## Introduction

HES1 (Hairy and enhancer of split 1) is a basic helix-loop-helix transcriptional factor that is expressed in the nuclei of normal intestinal epithelial cells and plays an important role in maintaining intestinal proliferative crypts and regulating enterocyte differentiation^1^. HES1 expression is regulated by the Notch pathway, a highly conserved pathway that regulates cellular proliferation and differentiation. In many tumors, aberrant Notch activation can contribute to cancer cell stemness, tumor cell proliferation, metastasis, and the reshaping of the tumor microenvironment^2–5^. Notch activation leads to the release of the Notch intracellular domain, which translocates to the nucleus and activates transcription of numerous downstream target genes, including *HES1*, *HES2, HEY1, HEY2,* and *DTX1*.

The precise role of HES1, a canonical Notch downstream transcription repressor, in intestinal carcinogenesis is controversial, with studies differing on the relationship between HES1 and colorectal cancer (CRC) outcomes. Although Weng et al. found that high expression of *HES1* mRNA correlated with poor prognosis^6^, Ahadi et al. used immunohistochemistry to demonstrate that loss of HES1 expression predicted worse prognoses in CRC patients^7^. These contrasting findings may be due to the presence of HES1 in the nuclei of both stromal cells and immune cells in cases where tumor cells are negative for HES1. Thus, studying HES1 in CRC progression using transcriptional expression may result in inconsistent findings. Alternatively, aberrant HES1 signaling may have distinct roles in different CRC pathways and in tumors with different genetic backgrounds.

HES1 may be related to CRC progression initiated by *KRAS* or *BRAF* mutations. In the canonical pathway of colorectal carcinogenesis, loss of the tumor suppressor *APC* (*adenomatous polyposis coli*) is followed by tumorigenic alterations of TP53, MAPK, and TGF-β signaling^8^. Therefore, the great majority of human CRCs, including hereditary syndromes and sporadic cancers, display *APC* mutations. However, CRC progression can be alternatively initiated by *KRAS* or *BRAF* mutations from adenomas with serrated morphologies^9,10^, and previous results suggest a relationship between HES1 and these pathways. Differentiation and proliferation of intestinal epithelium mediated by mutant *KRAS* was linked to activation of HES1, in a mouse model and human HP^11^. We reported that, on the contrary, loss of HES1 expression is observed in the majority of sessile serrated lesions (SSL) but not in hyperplastic polyps (HP)^12^. Moreover, we found that loss of HES1 expression is frequently observed in right-sided colon adenocarcinomas^13^, which commonly harbor *KRAS* or *BRAF* mutations^14^. Although most of the SSLs and the right-sided CRC with CpG island methylator phenotype (CIMP) arise from *BRAF* mutations, *KRAS* mutation is common in both CIMP-negative CRCs and CIMP-high but microsatellite stable (MSS) CRCs^15^.

To gain insight into the regulation between KRAS and HES1 in colorectal carcinogenesis, we examined microsatellite stable (MSS) and *KRAS* mutant CRCs that show aberrant expression of HES1 by immunohistochemistry. Using RNA sequencing and Nanostring’s RNA array analysis, we investigated differentially expressed genes and activated pathways regulated by HES1 in *KRAS* mutant CRCs.

## Material and methods

### Patients

The study of archived human CRC was approved by the Institutional Research Board (IRB) of the University Hospitals Case Medical Center, Cleveland Clinic Foundation, and Fudan University Shanghai Cancer Center. Informed consent was obtained from patients who agreed to donate tissues for the purpose of research according to the regulation by the IRB. All cases included in the study were confirmed as colorectal adenocarcinoma by two experienced pathologists. All methods used in this study were carried out in accordance with IRB guidelines and regulations. Demographic and clinicopathological data were collected from the medical records. Mutational status was determined by the ColonCore next-generation sequencing (NGS) panel (Burning Rock Biotech, Guangzhou, China) which is designed for simultaneous detection of microsatellite instability (MSI) status and mutations in 38 CRC related genes. Cases included in this study were microsatellite stable (MSS) CRCs that also had *KRAS* and *APC* mutation (Table 1). Another cohort of MSS and *KRAS* wild type CRCs were included. These cases were part of an IRB-approved annotated biobank. Biobank tumors had been previously evaluated for microsatellite instability and *KRAS* mutation status as previously described^15^. Cases that carry other frequently mutated genes (*TP53*, *BRAF*, *NRAS*) were excluded from the study.

**Table 1:** Patient demographics and tumor characteristics of *KRAS* mutant cohorts.

		HES1 (+) (n = 12)	HES1 (−) (n = 14)	*P* value
Age	≥ 60y	7 (58.3%)	10 (71.4%)	0.683
	< 60y	5 (41.7%)	4 (28.6%)	
Sex	Male	7 (58.3%)	9 (64.3%)	1.000
	Female	5 (41.7%)	5 (35.7%)	
Size	≥ 5 cm	4 (33.3%)	8 (57.1%)	0.267
	< 5 cm	8 (66.7%)	6 (42.9%)	
Location	Left	8 (66.7%)	10 (71.4%)	1.000
	Right	4 (33.3%)	4 (28.6%)	
Differentiation	Low grade	8 (66.7%)	11 (78.6%)	0.665
	High grade	4 (33.3%)	3 (21.4%)	
Infiltration depth	T1/T2	2 (16.7%)	2 (14.3%)	1.000
	T3/T4	10 (83.3%)	12 (85.7%)	
Lymph nodes	Positive	4 (33.3%)	7 (50.0%)	0.453
	Negative	8 (66.7%)	7 (50.0%)	
Distant metastasis	Positive	2 (16.7%)	6 (42.9%; 3 synchronous liver metastases)	0.216
	Negative	10 (83.3%)	8 (57.1%)	
KRAS	G12A	4 (33.3%)	4 (28.6%)	
	G12C	0 (0.0%)	2 (14.3%)	
	G12S	0 (0.0%)	1 (7.1%)	
	G12V	3 (25.0%)	4 (28.6%)	
	G13A	4 (33.3%)	2 (14.3%)	
	G61A	1 (8.3%)	0 (0.0%)	
	G61H	0 (0.0%)	1 (7.1%)	
APC	Frameshift mutation	10 (83.3%)	7 (50.0%)	
	Nonsense mutation	5 (41.7%)	11 (78.6%)	

### Gene expression analysis

Ten cases, 5 HES1 (−) and 5 HES1 (+), were subjected to RNA sequencing (cohort 1). RNA of these cases was isolated from the fresh frozen tissue followed by mRNA library preparation using Illumina’s TruSeq RNA Sample Prep Kit v2 (Illumina, RS-122-2001, San Diego, CA, USA). Sequencing was performed using Illumina HiSeq 2500 System (Illumina, San Diego, CA, USA). Another cohort of 9 cases, 4 HES1 (−) and 5 HES1 (+) were subjected to Nanostring RNA gene expression array analysis (cohort 2). RNA was isolated from formalin-fixed, paraffin-embedded (FFPE) tissue and assessed by the nCounter PanCancer IO 360 Panel (NanoString Technologies, Seattle, WA, USA)^16^.

### TCGA data acquisition

TCGA data of colorectal cancer (n = 431) level 3 gene-expression (counts) and somatic mutation were obtained from Genomic Data Commons (GDC) (https://portal.gdc.cancer.gov/). *KRAS* mutation was identified based on the “maftool” R package. The original counts data were transformed into transcript per kilobase million (TPM). Patients who lacked follow-up and somatic mutation information were excluded. A total of 155 colorectal cancer patients with *KRAS* mutation and 222 colorectal cancer patients with wild type *KRAS* were enrolled in this study. The high and low expression of HES1 in TCGA data was determined by the median expression (10.799) as a cutoff. Survival analysis was performed with Kaplan–Meier analysis in all colorectal cancer patients.

### Differentially expressed gene (DEG) and GSEA analysis

To identify genes associated with HES1 expression, DEGs was determined by using limma R package. The significant criteria were selected using *p* value < 0.05 and absolute fold-change (FC) > 1. The Venn Diagram was generated by the package of “venn”. Gene Set Enrichment Analysis (GSEA) analysis was performed by “ClusterProfiler” package.

### Immune cell infiltration analysis

Immune cells signature was determined by previously published method^17^. Briefly, Gene Set Variation Analysis (GSVA) was used to calculate the scale of value of each immune cell.

### Immunohistochemical staining and evaluation

Paraffin blocks of 25 cases from cohort 1 and 2 were selected for the construction of the tissue microarray (TMA). For each block, three cores with a diameter of 2 mm were obtained from the tumor. Immunohistochemical staining (IHC) was performed using the automated immunostainer (Ventana, Tucson, AZ, USA). Primary antibodies used in this study include HES1 (Clone: EPR4226, Cat. No. ab108937, Abcam), Ki67 (Clone: 30-9, Cat. No. 790-4286, Ventana), TP53 (Clone: DO-7, Cat. No. M7001, Dako), RB1 (Clone: 4H1, Cat. No. 9309, Cell Signaling Technology), Cyclin D1 (Clone: SP4-R, Cat. No. 790-4508, Ventana), E-cadherin (Clone: NCH-38, Cat. No. M3612, Dako), Vimentin (Clone: V9, Cat. No. IR630, Dako), CD44 (Clone: DF1485, Cat. No. M7082, Dako), CD8 (Clone: SP57, Cat. No. 790-4460, Ventana), CD163 (Clone: MX081, Cat. No. MAB-0869, Fuzhou Maixin Biotechnology), CD68 (Clone: KP1, Cat. No. M-0160-1.0, Shanghai Changdao Biotechnology), phospho-STAT3 (Tyr705, Clone: D3A7, Cat. No. 9145, Cell Signaling Technology), IL10 (Clone: 2472A, Cat. No. MAB91842, R&D Systems). Expression of HES1 was evaluated as previously described^12^. The presence of HES1 nuclear expression was considered HES1 (+), while loss of HES1 nuclear expression was classified as HES1 (−). Histoscores (H-scores) were calculated by multiplying the staining intensity (0 = negative, 1 = weak, 2 = moderate, 3 = strong) and the percentage of positive cells (number of positive tumor cells/ number of total tumor cells, range 0–100). All cases were scored by two experienced pathologists. The expression status of TP53, RB1 and Cyclin D1, IL10 were evaluated using H-scores. Tumor cells showed homogeneously strong membrane expression of E-cadherin were considered positive, while weak or loss expression of E-cadherin of tumor cells was classified as abnormal. The percentage of positive Ki67 staining in tumor cells was evaluated. Densities of CD8, CD163 and CD68 were calculated (area of positive immune cells/total area of tissue).

### Cell culture, shRNA knockdown, immunoblotting, MTT and tumor migration analysis

CRC cells were obtained from AATC (Manassas, VA) with certified characterization and cultured according to the instructions. HES1 knockdown was performed in CRC cells transfected with four shRNA against HES1 (TG312478) or 29-mer scrambled shRNA as negative control (TR30013) (OriGene, Rockville, MD). Cells were harvested 72 h post-transfection. Western blot was performed to assess knockdown efficiency. Membranes were pre-cut prior to hybridization with primary antibodies including HES1 (Clone: D6P2U, Cat. No. 11988, Cell Signaling Technology), IL10 (Clone: 1O10, Cat. No. ZRB2535, Sigma-Aldrich) followed by imaging analysis using the ChemiDoc Touch Imaging System (Bio-Rad) and the Image Lab 6.1 Software for Windows (Bio-Rad). MTT and wound healing assays were performed as described^18^. qRT-PCR was performed as described^19^. All primer sequences are provided upon request.

### Statistical analysis

Data were analyzed using R software (version4.2.1). Comparisons of ≥ 2 groups were conducted using a parametric test (Student t-test or ANOVA test) or a nonparametric test (Wilcoxon rank-sum test or Kruskal–Wallis test, Pearson Chi-Square test or Fisher’s exact test). ns, *, **, and *** represent not significant (*p* ≥ 0.05), *p* < 0.05, *p* ≤ 0.01, and *p* ≤ 0.001, respectively.

### Ethical approval and consent to participate

The study of archived human CRC was approved by the Institutional Research Board of the University Hospitals Case Medical Center, Cleveland Clinic Foundation, and Fudan University Shanghai Cancer Center. Informed consent was obtained from patients who agreed to donate tissues for the purpose of research.

## Results

### Identification of biological pathways correlated with HES1-loss in *KRAS* mutant CRC

**Figure 1:**
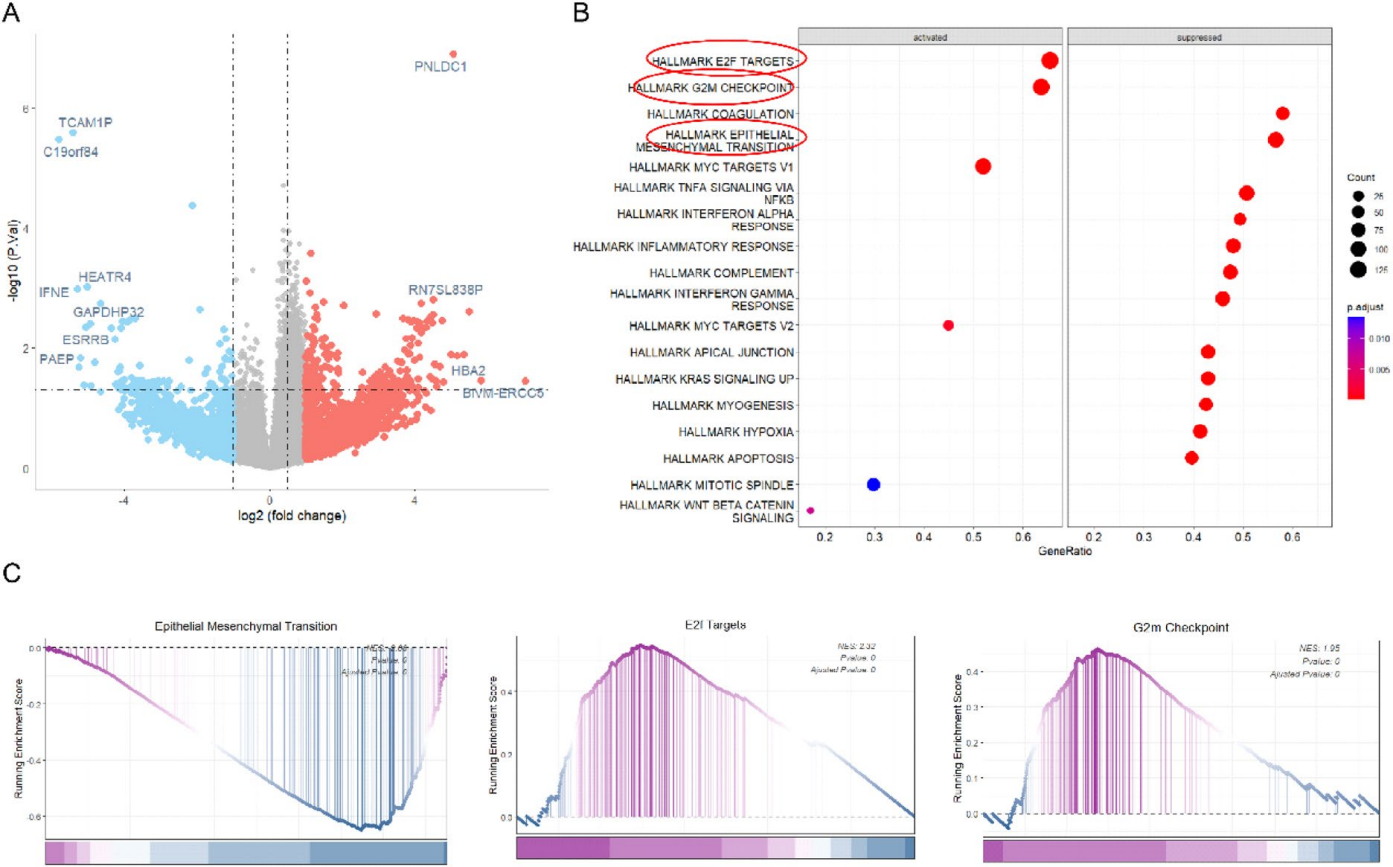
RNA sequencing analyses. (**A**) A total of 360 DEGs were obtained between HES1 (+) group (n = 5) and HES1 (−) group (n = 5) (*p* < 0.05), of which 248 DEGs were downregulated and 112 DEGs were upregulated in HES1 (−) group. (**B**) The biological pathways implicated by the aberrant HES1 expression revealed by GSEA analysis of the DEGs of HES1 (+) and HES1 (−) groups. (**C**) GSEA plots showing that HES1 loss was positively correlated with “EPITHELIAL_MESENCHYMAL_TRANSITION” signaling, but negatively correlated with “E2F_TARGETS” and “G2M_CHECKPOINT” signaling.

We found that loss of HES1 nuclear expression is more frequently associated with CRCs harboring *BRAF* or *RAS* mutations^13,14^. To understand the reciprocal regulation between KRAS and HES1, we examined the HES1 expression in *KRAS* mutant CRCs. These *KRAS* mutant cases (Table 1) were sequenced by the ColonCore next-generation sequencing panel. We selected 5 cases with HES1 nuclear expression, referred as HES1 (+), and 5 cases with loss of HES1 nuclear expression, referred as HES1 (−) (Fig. S1), in cohort 1. All cases had *KRAS* and *APC* mutation while other frequently mutated genes were wild type (Tale S1). In addition, all ten cases were determined to be MSS. RNA sequencing of this cohort revealed 360 differentially expressed genes (DEGs), of which 248 were significantly downregulated while 112 were upregulated in the HES1 (−) group (Fig. 1A). To investigate the biological pathways implicated by aberrant HES1 expression, we subjected these DEGs to GSEA. We found that HES1-loss positively correlated with EPITHELIAL_MESENCHYMAL_TRANSITION but negatively correlated with E2F_TARGETS and G2M_CHECKPOINT (Fig. 1B,C).

**Figure 2:**
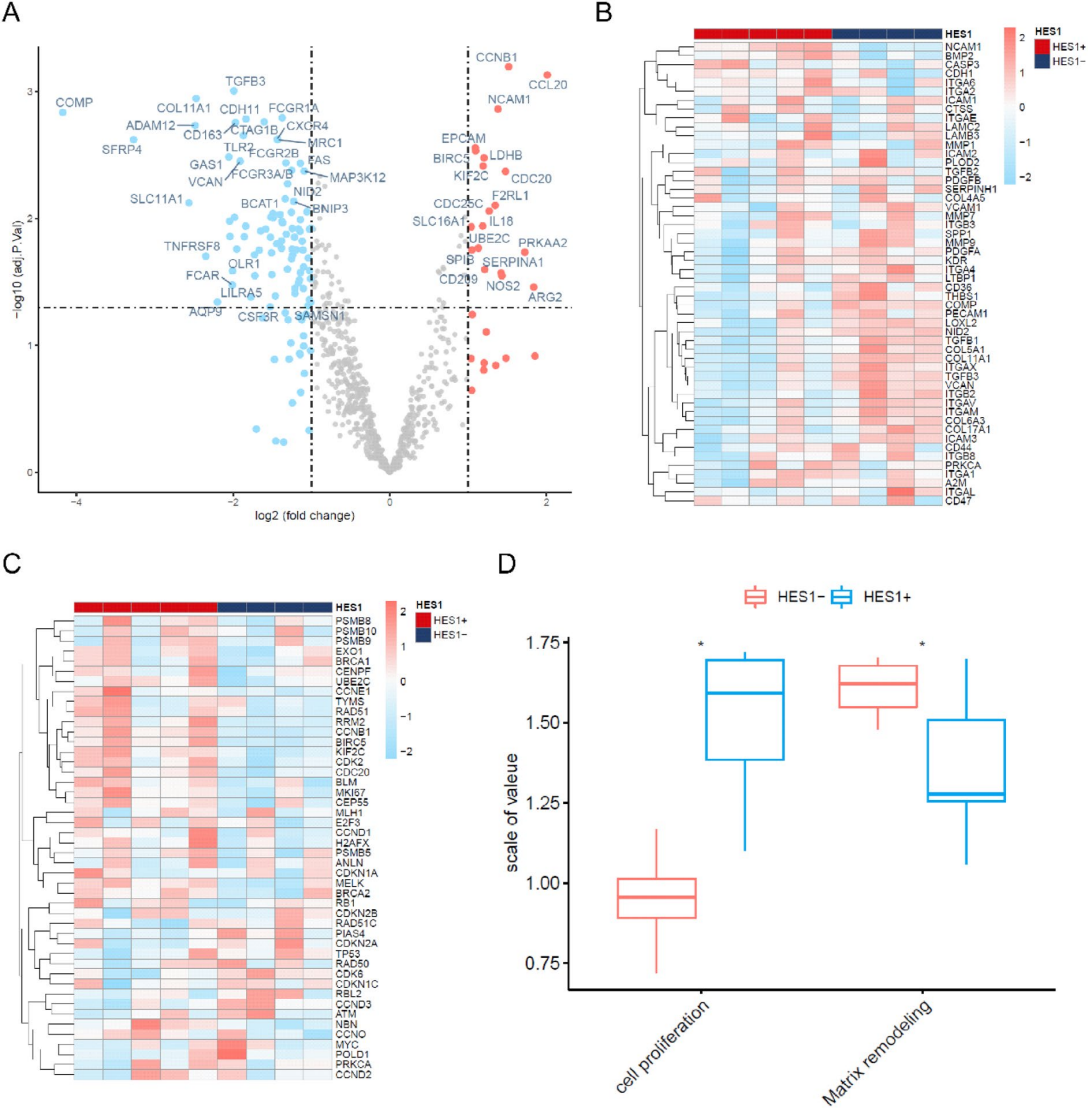
Nanostring RNA gene expression array analyses. (**A**) Volcano plot identified 93 DEGs between HES1 (+) (n = 5) and HES1 (−) group (n = 4) (*p* < 0.05), of which 82 DEGs were upregulated and 19 DEGs were downregulated in HES1 (−) group. (**B**) A heatmap of 52 matrix remodeling and metastasis process-related genes (rows) is shown for 9 samples (columns) including HES1 (−) (blue bar) and HES1 (+) cases (red bar). (**C**) A heatmap of 46 differentially expressed genes included in the cell proliferation process (rows) for 9 samples (columns) including HES1 (−) (blue bar) and HES1 (+) cases (red bar). (**D**) The GSVA plot showing that loss of HES1 was positively correlated with the matrix remodeling and metastasis process and negatively correlated with cell proliferation process (*p < 0.05).

To verify these findings, we selected another cohort of nine cases (cohort 2) including 5 HES1 (+) and 4 HES1 (−). We examined the transcriptional profile of these cases using the NanoString nCounter PanCancer IO 360 panel, which profiles 750 cancer-related human genes across 16 key immuno-oncology pathways both within the tumors and at the interface of tumor stroma interaction and tumor immune responses. All these cases were MSS and had mutations in *KRAS* and *APC*. Other frequently mutated genes were wild type (Table S2). Of the 93 DEGs found, 19 were downregulated and 82 upregulated in the HES1 (−) group compared to the HES1 (+) group (Fig. 2A). HES1-loss positively correlated with matrix remodeling and metastasis (Fig. 2B) and negatively correlated with cell proliferation (Fig. 2C,D).

To assess if these differentially regulated signaling pathways are uniquely associated with *KRAS* mutation, we selected a cohort of *KRAS* wild type (WT) CRC cases (cohort 3), composed of 6 HES1 (+) and 6 HES1 (−) samples. Analysis of the transcriptional profile using the NanoString nCounter PanCancer IO 360 panel identified 12 DEGs between the HES1 (+) (n = 6) and the HES1 (−) group (n = 6), of which 6 DEGs were upregulated and 6 downregulated in the HES1 (−) group. Among these, *LAMA1* (Laminin Subunit Alpha 1), which encodes the extracellular matrix glycoprotein, displayed a higher expression in HES1 (−) group than in HES1 (+) group. However, unlike *KRAS* mutant CRCs, matrix remodeling, EMT, and cell proliferation were not associated with HES1-loss in *KRAS* WT CRCs (Fig. S2).

### Differential EMT marker and proliferation marker expression is regulated by HES1 in *KRAS* mutant CRC

**Figure 3:**
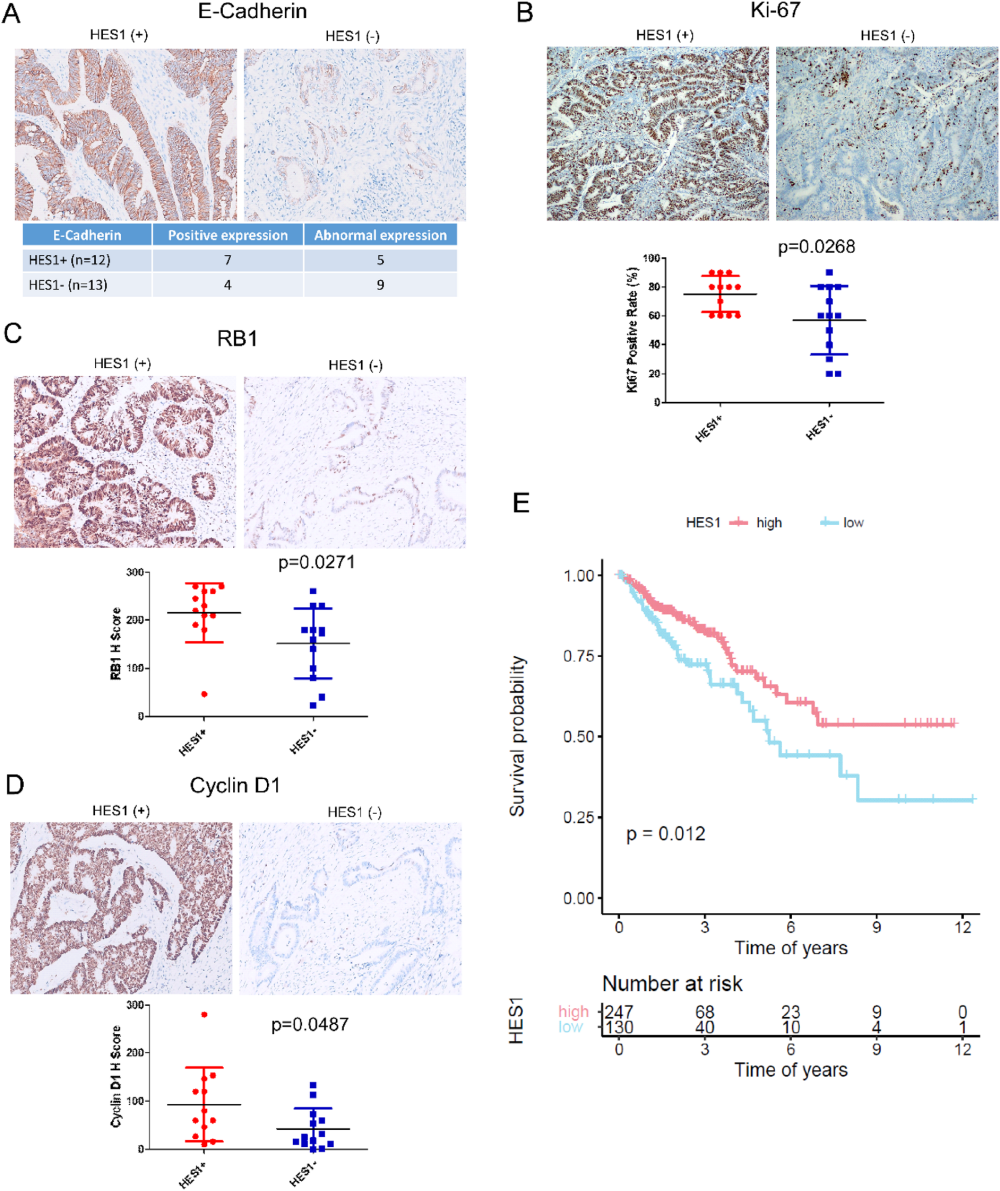
Expression of EMT and proliferation markers. (**A**) Representative immunohistochemistry staining of E-cadherin and the analysis of its aberrant expression associated with HES1 (−) CRCs. (**B**–**D**) Representative immunohistochemistry staining of Ki67 (**B**), RB1 (**C**) and Cyclin D1 (**D**). Differential IHC scores were shown below. (**E**) Poor prognosis associated with low HES1 expression in TCGA CRC dataset.

Results from NanoString analysis showed positive correlation between HES1-loss and tumor migration and invasion but negative correlation with tumor proliferation. We assessed the expression of EMT markers such as E-cadherin, Vimentin and CD44, finding that HES1 (−) CRCs more frequently show weak or negative staining of E-cadherin compared to HES1 (+) CRCs. Weak expression or no expression of E-cadherin is observed in 69.2% (9/13) of the HES1 (−) group but in 41.7% (5/12) of the HES1 (+) group (Fig. 3A). There is no significant difference in the expression of Vimentin or CD44 between these two groups (data not shown). However, three HES1 (−) cases exhibited liver metastases identified upon CRC diagnosis (Table 1), suggesting a more rapid progression of CRCs compared to HES1 (+) cases, where no liver metastases were found.

We also assessed the expression of the cell cycle related markers (Ki67, TP53, RB1 and Cyclin D1). Compared to HES1 (−) tumors, expression levels of Ki67 (*p* = 0.0268) (Fig. 3B), RB1 (*p* = 0.0271) (Fig. 3C) and Cyclin D1 (*p* = 0.0487) (Fig. 3D) were all significantly upregulated in the HES1 (+) tumor cells. TP53 (*p* = 0.2664) had a trend of increased expression in the HES1 (+) group (data not shown). Corroborating RNA sequencing and transcriptional profiling by RNA array, analysis of TCGA CRC data set identified a worse prognosis in patients who have lower expression of HES1 (Fig. 3E).

**Figure 4:**
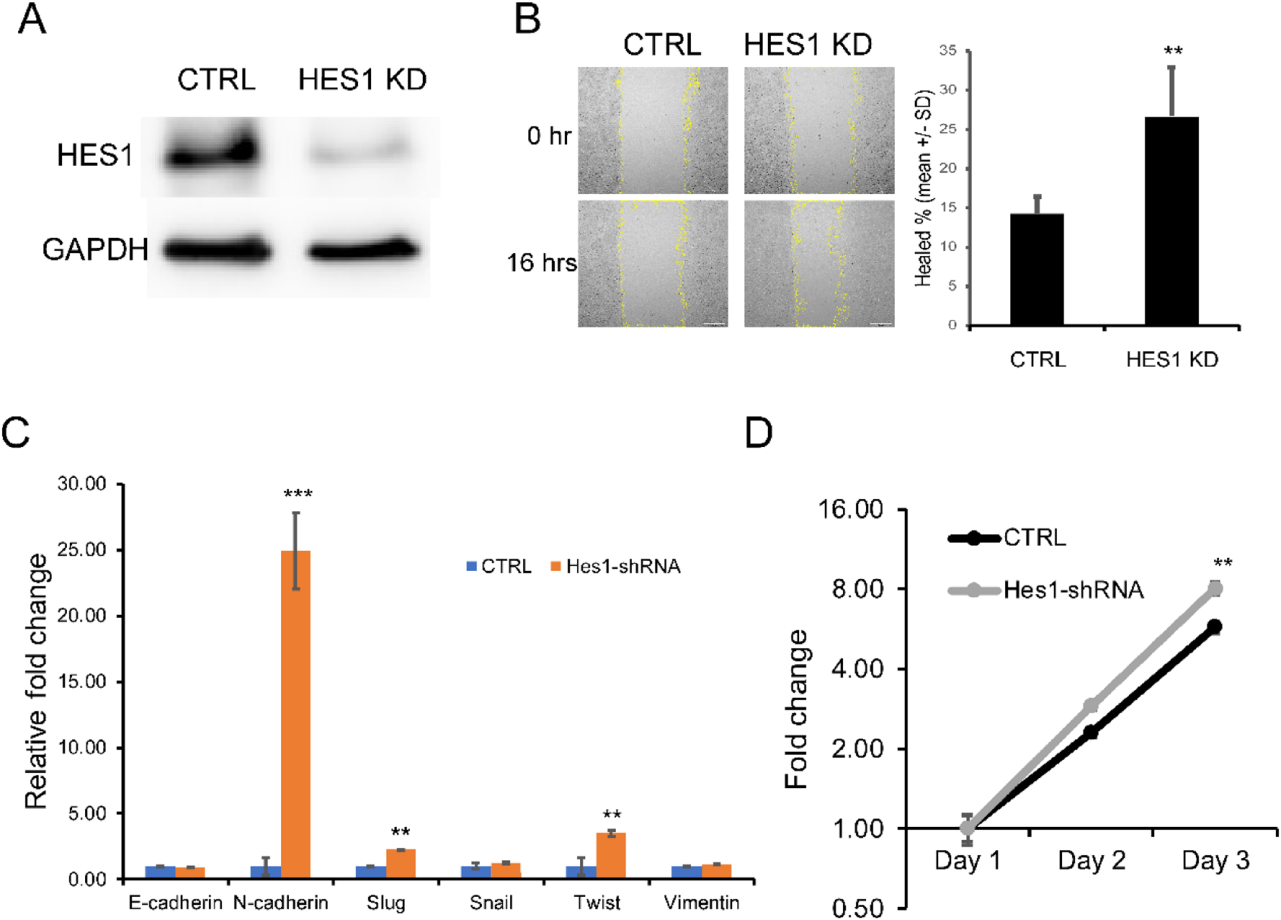
HES1 downregulation increased CRC cell migration and EMT marker expression. (**A**) HES1 expression in SW620 cells decreased by HES1 shRNA (HES1 KD). (**B**) Migration assays showing the decrease of cell migration in HES1 KD cells. (**C**) qRT-PCR showing expression of EMT markers in HES1 KD cells vs controls. (**D**) Increased cellular proliferation in HES1 KD cells by MTT assays. Data shown in (**B**–**D**) represents the means ± SD of three to six independent experiments. **p < 0.01; ***p < 0.001.

To assess if HES1-loss regulates cellular proliferation, invasion and EMT, we selected SW620 CRC cell line whose mutation and MSI status match those of human specimens we examined. We found that knocking down of *HES1* increased cell migration by wound healing assay (Fig. 4A,B and Fig. S3A) but had no impact on cell cycling (not shown). The expression of EMT markers assessed by qRT-PCR revealed increased mRNA levels of *CDH2*, *TWIST* and *SLUG*. Expression of *CDH1* slightly decreased (Fig. 4C). We found that SW620 cell proliferation increased upon HES1 downregulation (Fig. 4D). Therefore, induced HES1-loss in vitro promoted tumor invasion and EMT. However, a negative correlation between HES1 and cellular proliferation was not recapitulated under the in vitro culture conditions.

### HES1-loss correlates with higher M2 macrophage signature in *KRAS* mutant CRC

**Figure 5:**
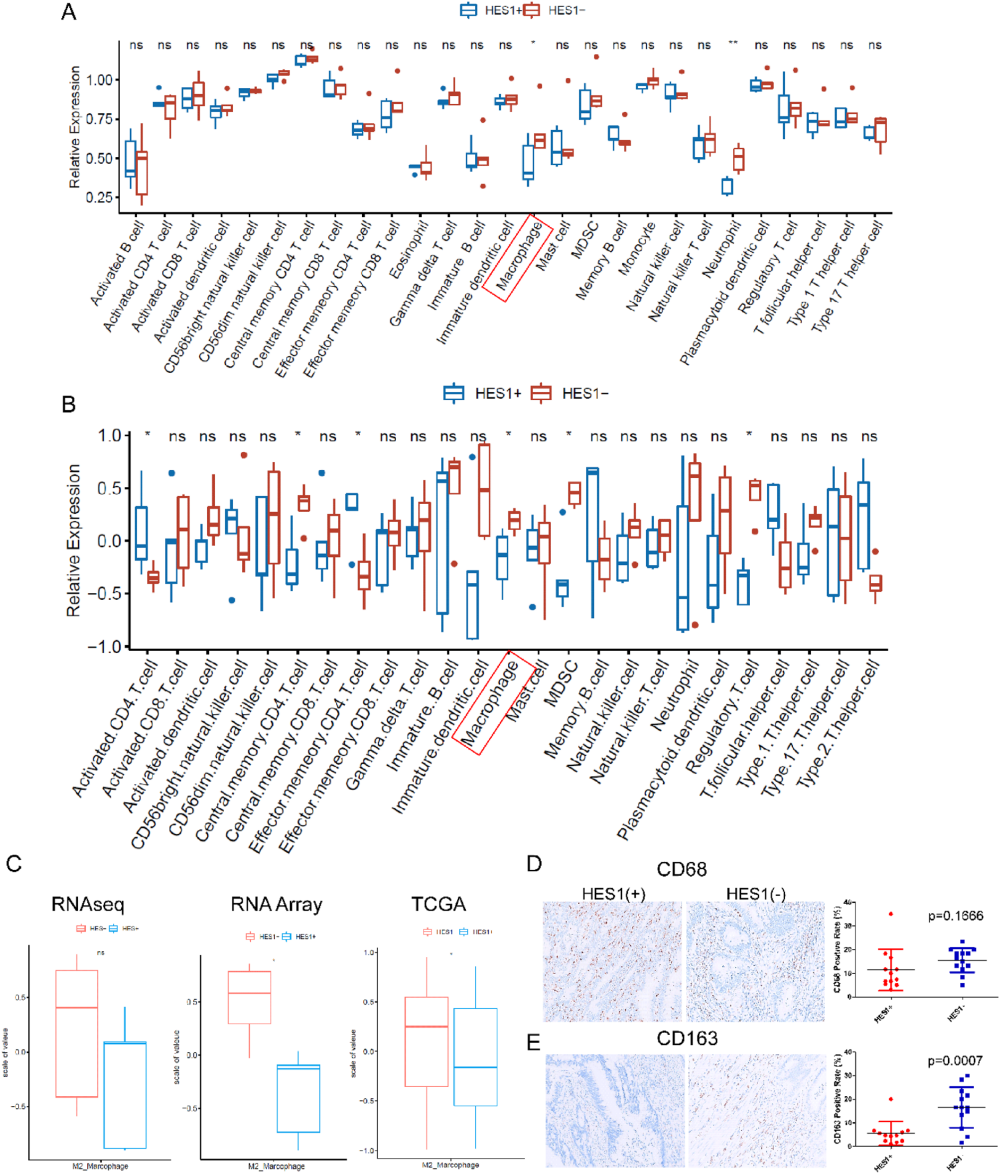
Tumor infiltrating immune cell signatures and macrophage markers in HES1 (+) and HES1 (−) CRCs. (**A**, **B**) Tumor infiltrating immune cell signature was analyzed. The signature of macrophage was elevated in HES1 (−) group from both RNA sequencing (**A**) and nanostring array (**B**) (*p* < 0.05). M2 macrophage were sub-clustered by the expression of CD206, CD204 and CD163. Higher M2 macrophages in HES1 (−) group were observed in RNA sequencing, nanostring array and TCGA data (*p < 0.05) (**C**). A pan-macrophage or M1 macrophage marker, CD68 (**D**), and the M2 macrophage marker, CD163 (**E**) was examined by IHC, both of which showed cytoplasmic staining. There was no significant difference in the density of CD68 positive macrophages between HES1 (+) and HES1 (−) groups (*p* = 0.1666) (**D**). The density of CD163 positive macrophages was much higher in HES1 (−) group (*p* = 0.0007) (**E**).

A significant set of genes enriched by HES1 (−) CRCs are related to inflammatory pathways and responses, including TNFα signaling via NFKB, the inflammatory response, the interferon α response, and the interferon γ response (Fig. 1B). Thus, we evaluated tumor infiltrating immune cells in HES1 (+) and HES1 (−) groups. Higher density of macrophage infiltration in HES1 (−) group was found by both RNA sequencing and NanoString Array (Fig. 5A,B). In the HES1 (−) group, RNA sequencing found higher neutrophil density in the HES1 (−) group, while NanoString Array analysis found upregulation of regulatory CD4 T cells, myeloid-derived suppressive cells (MDSCs), and regulatory T cells. M2 macrophages were sub-clustered according to the expression of genes including CD206, CD204, and CD163^20^. Both RNA sequencing and the NanoString array found that genes expressed by M2 macrophages were higher in the HES1 (−) group than in the HES1 (+) group. Consistently, analysis of M2 macrophage gene expression in the TCGA data set showed an increase of M2 macrophage gene expression in patients who have lower expression of HES1 (Fig. 5C).

We confirmed the results from RNA sequencing and NanoString array with IHC. CD68-positive or CD163-positive macrophages were mainly detected in the tumor stroma. While CD68 is normally considered a pan-macrophage or M1 macrophage marker, CD163 is accepted as a M2 macrophage marker^21,22^. We found no obvious difference in the density of CD68-expressing macrophage between the HES1 (+) and HES1 (−) groups (*p* = 0.179) (Fig. 5D). However, the density of CD163-positive macrophage was much higher in the HES1 (−) group than in the HES1 (+) group (*p* = 0.0007) (Fig. 5E) confirming transcriptome profiling analysis.

### Signaling pathways in IL6 and IL10 correlates with higher M2 macrophage in HES1-loss *KRAS* mutant CRC

**Figure 6:**
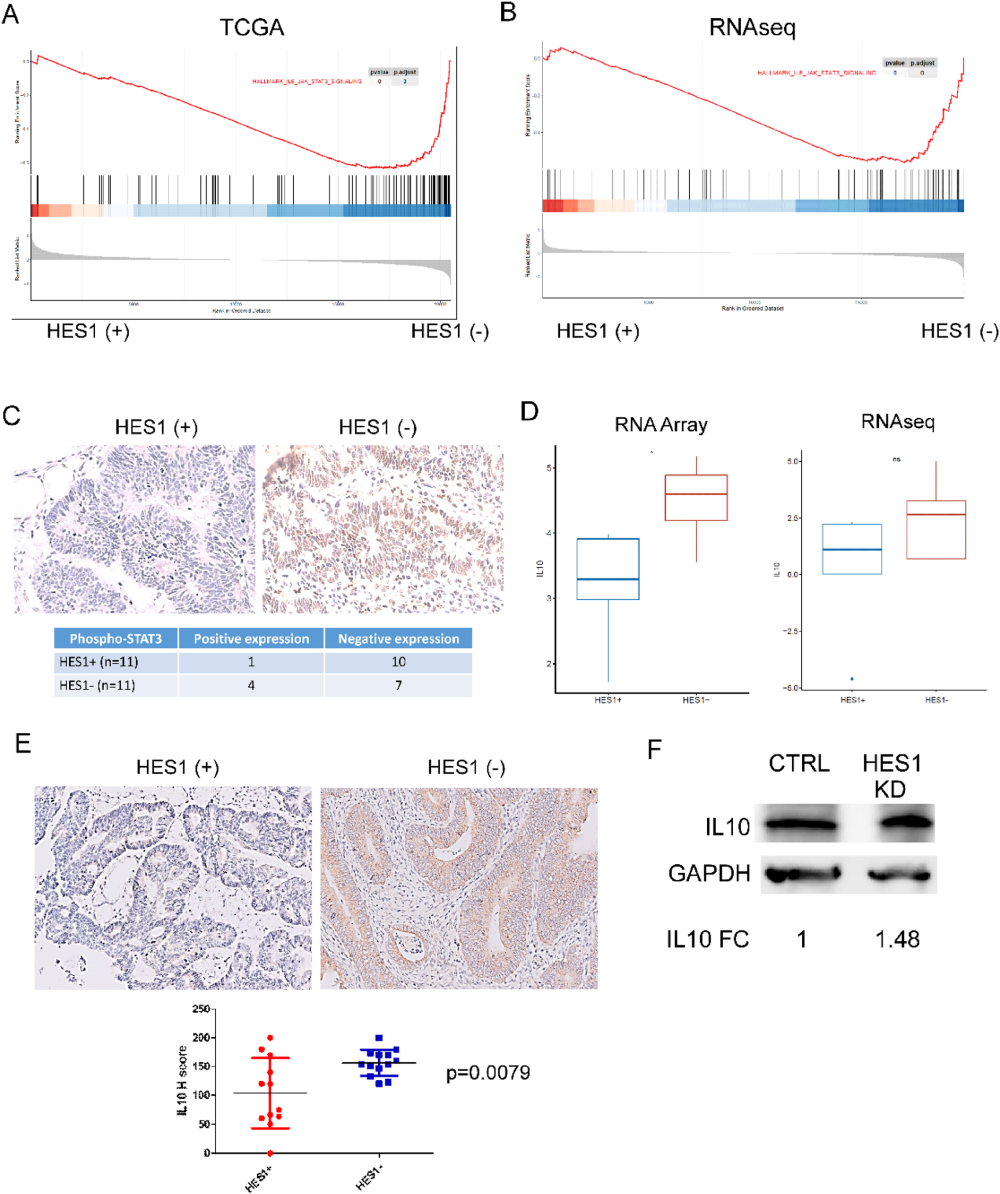
Signaling pathways involved in higher M2 macrophage in HES1-loss *KRAS* mutant CRC. (**A**, **B**) “IL6_JAK_STAT3” signaling activation in HES1(−) group was identified by GSEA analysis of TCGA data set (**A**) and RNA sequencing (**B**). (**C**) The expression of phospho-STAT3 was evaluated by IHC, which displayed nuclear staining. Numbers of phospho-STAT3 (+) cases was higher in HES1 (−) group than in HES1 (+) group. (**D**, **E**) Expression of M2 macrophage related cytokine, IL10, was assessed by RNA array (*p < 0.05) and RNA sequencing (**D**) as well as by IHC (**E**). (**F**) IL10 protein expression was assessed by Western blot in SW620 control and HES1 KD cells and relative fold change (FC) was shown. Data shown was a representative of three similar experiments.

To explore the significance of inflammatory response and tumor-associated macrophage (TAM) linked to HES1-loss in *KRAS* mutant CRC in a larger database, we performed GSEA analysis of HES1-high and HES1-low *KRAS* mutant CRC cases in the TCGA dataset. GSEA analysis found that HES1-low positively correlated with IL6_JAK_STAT3_SIGNALING (Fig. 6A). A similar signature was observed from RNA sequencing analysis (Fig. 6B). IL6 secreted by TAMs promotes CRC proliferation and invasion through IL6/STAT3 signaling^23^. We found that tumor cells in 36.4% (4/11) cases of HES1 (−) group were positive for phospho-STAT3 while only 9.1% (1/11) of HES1 (+) cases were positive for phospho-STAT3 (Fig. 6C) but statistical significance was not reached.

We then examined other cytokines associated with HES1-loss that may potentiate M2 macrophage accumulation. NanoString Array analysis found that IL10 mRNA expression was upregulated in the HES1 (−) group. RNA sequencing also revealed a positive trend of IL10 expression in the HES1 (−) group (Fig. 6D). Using IHC, we found that IL10, which polarizes macrophages towards the M2 phenotype^24^, shows expression by tumor cells. Overall, IL10 expression in the HES1 (−) group was higher than in the HES1(+) group (*p* = 0.0079) (Fig. 6E). Our results thus suggest that IL10 released from HES1 (−) CRC tumors into the TME may play a role in M2 macrophage polarization. Corroborating tissue studies, we found that SW620 cells increased expression of IL10 upon HES1 down-regulation (Fig. 6F, Fig. S3B).

## Discussion

The role of Notch signaling in colorectal carcinogenesis and progression remains controversial. Reports have shown that Notch activation and Wnt signaling act synergistically to promote the initiation of adenoma formation^25^. Notch activation by copy number gain of *NOTCH1* is associated with a worse clinical prognosis^26^. Activated Notch signaling combined with additional oncogenic driver mutations also drive CRC invasion and metastasis in animal models^27,28^. Further, reports have shown that Notch signaling activation and *KRAS* mutation is significantly associated with poor prognosis in human CRC^27^. On the contrary, our group found that nuclear HES1 expression is lost in 91% of sessile serrated adenomas/polyps (SSA/p) and most of the right-sided colorectal cancer which commonly harbors *BRAF* or *KRAS* mutation^12,13^. It has been proposed that *KRAS* mutation creates a subset of CRCs that arises via the serrated pathway^29^. However, the mechanisms of aberrant Notch signaling in the progression of human CRC and its cross-regulation with KRAS are still unclear.

In this study, we focused on the MSS CRCs that carry *KRAS* mutation to assess how aberrant HES1 expression impacts genes and pathways that may affect CRC tumorigenesis and progression. Because most of the *KRAS* mutant CRCs also have *APC* mutation, we included both in our study cohorts and classify these cases into HES1 (+) and HES1 (−) groups according to the presence or absence of tumor nuclear expression of HES1. By using two different transcriptome profiling approaches, we identified commonly affected pathways regulated by HES1, a canonical target of Notch signaling.

Our work revealed that loss of HES1 expression positively correlated with matrix remodeling and epithelial-mesenchymal transition (EMT) but negatively correlated with tumor cell proliferation. These findings indicated that absence of nuclear HES1 expression suppresses tumor cell proliferation but also promotes CRC invasion. Uncontrolled proliferation and invasion are the dominant characteristics of malignant tumors^30^. However, these two cellular processes do not always occur simultaneously, a phenomenon described as migration-proliferation dichotomy^31,32^. Indeed, we found that loss of HES1 correlated with decreased E-cadherin expression in CRC. Decreased expression of E-cadherin on epithelial cell surface is a crucial marker of EMT process. Consistently, we found liver metastases in 25% of patients whose colon lesions lost HES1 expression when diagnosed. On the other hand, loss of nuclei HES1 CRC cases showed decreased expression of cell cycling markers including Ki67, Cyclin D1, RB1 and TP53. Therefore, HES1 likely functions as a migration-proliferation dichotomy node that controls tumor invasion and proliferation in *KRAS* mutant CRC, and its loss may be a predictor of tumor invasion and metastasis. While CRC cells harboring KRAS mutation and expressing higher levels of HES1 displayed increased migration and altered EMT marker expression upon *Hes1* down-regulation, inhibition on cellular proliferation by HES1 downregulation was not observed. These findings suggest that HES1-loss may collaborate with other pathways to regulate CRC proliferation in vivo. Supporting this note, tumor mutation profiling of HES1-low tumors from TCGA dataset showed a higher mutational frequency in several genes such as *TP53*, *FAT4*, and *AMER1* (Fig. S4).

Interestingly, we did not find the correlation between HES1 expression and EMT process and cell proliferation in *KRAS* WT CRCs. A correlation with *KRAS* mutant CRC suggests that aberrant HES1 expression may interact with RAS signaling to promote invasion and metastasis. Few studies have focused on the relationship between HES1 expression and *KRAS* mutations. Feng et al. found that, in a mouse model, mutant Kras mediated colon epithelium differentiation and proliferation was linked to activation of Hes1^11^. Kim et al.^27^ investigated the clinical significance of HES1 expression in human small intestinal adenocarcinomas. Consistent with our findings, patients with *KRAS* mutant tumors that showed loss of HES1 expression had worse prognoses. This study also reported independence between the prognosis of patients with positive HES1 expression and *KRAS* mutation status. We recently found that loss of HES1 expression in CRC was associated with *KRAS* or *BRAF* mutation while almost all the *KRAS/BRAF* mutant tumors located on the right colon show negative HES1 expression^14^. However, the exact mechanism linking aberrant HES1 expression to *KRAS/BRAF* mutant tumor invasion and metastasis remains unknown.

Our results also suggest that alteration of HES1 expression in tumor cells can rewire the tumor microenvironment and affect tumor progression through M2 macrophage polarization. Macrophages play a crucial role in tumor immune microenvironment. While M1-like macrophages are commonly referred to as pro-inflammatory and anti-tumoral, M2 macrophages, marked by CD163 and CD206, often present anti-inflammatory and immunosuppressive activities^33^. Tumor-associated macrophages (TAMs) in advanced tumors often closely resemble the M2-like macrophages and can exert immunosuppression in the tumor microenvironment^34^. TAMs of CRC, polarized to M2-like phenotype by cytokines such as IL4, IL13, IL10, produce anti-inflammatory cytokines including TGFβ and IL10 and are associated with a poor prognosis^35,36^. We found that HES1 (−) CRCs had higher density of CD163-positive macrophages and displayed higher level of IL10 when compared to HES1 (+) tumors. While myeloid derived suppressor cells are likely the major source of IL10 in CRC^37^, we found that HES1 downregulation in CRC cells increased IL10 expression suggesting a potential tumor-intrinsic role of IL10 in the pathogenesis of HES1 (−) CRCs. HES1 can directly regulate IL10 since the promoter region of IL10 contains an HES1 N-box binding sequence. However, IL10 increase in HES1 (−) tumors may implicate the complex tumor microenvironment remodeled by KRAS mutation and/or HES1-loss.

Factors produced by the immune cells, stromal cells, and cancer cells regulate all aspects of tumor pathogenesis and progression. IL6, for example, may function as a critical link between inflammation and CRC development^38^. Other studies have shown IL6 polarizes M2 macrophage in CRC^39^ and that IL6/STAT3 can form a positive feedback loop to stimulate tumor growth and progression^40^. The exact mechanism and impact of enhanced IL6/STAT3 signaling in HES1 (−) CRC warrants further investigation. Nevertheless, these findings support a vital role of M2 macrophage polarization and a role of IL10 and IL6/STAT3 signaling in HES1 (−) *KRAS* mutant CRCs that may act in concert to promote tumor progression and metastasis. Finally, RNA array revealed upregulation of MDSCs and regulatory T cells in HES1 (−) CRCs, suggesting these immune cells may also promote HES1 (−) CRC tumorigenesis.

*KRAS* mutations are associated with decreased response rate to anti-EGFR therapy^41,42^. Inhibitors that selectively target KRAS G12C is promising but this type of inhibitors has limited mutation targets^43^. Most CRC patients, except those whose tumors have high levels of MSI or are deficient in mismatch repair, cannot benefit from FDA-approved immune check point inhibitors^44,45^. Our findings identify signaling pathways and cytokines impacted by HES1 that are responsible for promoting tumor progression in *KRAS* mutant CRCs. These markers may be useful for prognostic prediction and future design of novel therapeutics for *KRAS* mutant CRCs.

## Conclusion

In summary, our study indicates that aberrant HES1 expression correlates with tumor matrix remodeling in *KRAS* mutant CRC. Loss of HES1 also plays a role in rewiring the tumor immune microenvironment to induce immune suppression.

### Supplementary Information

Supplementary Information.

## Data Availability

The datasets generated and/or analysed during the current study are available in the Genome Sequence Archive (Genomics, Proteomics & Bioinformatics 2021) in National Genomics Data Center (Nucleic Acids Res 2022), China National Center for Bioinformation/Beijing Institute of Genomics, Chinese Academy of Sciences (GSA-Human: HRA003857) that are publicly accessible [https://bigd.big.ac.cn/gsa-human/browse/HRA003857]. Data and material are available upon request.

## References

[1] Kopan R, Ilagan MX (2009). The canonical Notch signaling pathway: Unfolding the activation mechanism. Cell.

[2] Lu J, Ye X, Fan F, Xia L, Bhattacharya R, Bellister S (2013). Endothelial cells promote the colorectal cancer stem cell phenotype through a soluble form of Jagged-1. Cancer Cell.

[3] Sonoshita M, Itatani Y, Kakizaki F, Sakimura K, Terashima T, Katsuyama Y (2015). Promotion of colorectal cancer invasion and metastasis through activation of NOTCH-DAB1-ABL-RHOGEF protein TRIO. Cancer Discov..

[4] Rodilla V, Villanueva A, Obrador-Hevia A, Robert-Moreno A, Fernández-Majada V, Grilli A (2009). Jagged1 is the pathological link between Wnt and Notch pathways in colorectal cancer. Proc. Natl. Acad. Sci. U. S. A..

[5] Wieland E, Rodriguez-Vita J, Liebler SS, Mogler C, Moll I, Herberich SE (2017). Endothelial Notch1 activity facilitates metastasis. Cancer Cell.

[6] Weng MT, Tsao PN, Lin HL, Tung CC, Change MC, Chang YT (2015). Hes1 increases the invasion ability of colorectal cancer cells via the STAT3-MMP14 pathway. PLoS ONE.

[7] Ahadi M, Andrici J, Sioson L, Sheen A, Clarkson A, Gill AJ (2016). Loss of Hes1 expression is associated with poor prognosis in colorectal adenocarcinoma. Hum. Pathol..

[8] Fearon ER, Vogelstein B (1990). A genetic model for colorectal tumorigenesis. Cell.

[9] Jass JR (2001). Serrated route to colorectal cancer: Back street or super highway?. J. Pathol..

[10] Yang S, Farraye FA, Mack C, Posnik O, O'Brien MJ (2004). BRAF and KRAS mutations in hyperplastic polyps and serrated adenomas of the colorectum: Relationship to histology and CpG island methylation status. Am. J. Surg. Pathol..

[11] Feng Y, Bommer GT, Zhao J, Green M, Sands E, Zhai Y (2011). Mutant KRAS promotes hyperplasia and alters differentiation in the colon epithelium but does not expand the presumptive stem cell pool. Gastroenterology.

[12] Cui M, Awadallah A, Liu W, Zhou L, Xin W (2016). Loss of Hes1 differentiates sessile serrated adenoma/polyp from hyperplastic polyp. Am. J. Surg. Pathol..

[13] Wang Y, Huang D, Chen KY, Cui M, Wang W, Huang X (2017). Fucosylation deficiency in mice leads to colitis and adenocarcinoma. Gastroenterology.

[14] Chen W, Zhou L, Xin W (2022). High frequency of HES1 loss in colorectal adenocarcinomas with RAS/BRAF mutations. J. Clin. Transl. Pathol..

[15] Sanchez JA, Krumroy L, Plummer S, Aung P, Merkulova A, Skacel M (2009). Genetic and epigenetic classifications define clinical phenotypes and determine patient outcomes in colorectal cancer. Br. J. Surg..

[16] Lal JC, Townsend MG, Mehta AK, Oliwa M, Miller E, Sotayo A (2021). Comparing syngeneic and autochthonous models of breast cancer to identify tumor immune components that correlate with response to immunotherapy in breast cancer. Breast Cancer Res..

[17] Gu W, Kim M, Wang L, Yang Z, Nakajima T, Tsushima Y (2021). Multi-omics analysis of ferroptosis regulation patterns and characterization of tumor microenvironment in patients with oral squamous cell carcinoma. Int. J. Biol. Sci..

[18] Yu S, Parameswaran N, Li M, Wang Y, Jackson MW, Liu H (2017). CRABP-II enhances pancreatic cancer cell migration and invasion by stabilizing interleukin 8 expression. Oncotarget.

[19] Wang Y, Yu S, Huang D, Cui M, Hu H, Zhang L (2016). Cellular prion protein mediates pancreatic cancer cell survival and invasion through association with and enhanced signaling of Notch1. Am. J. Pathol..

[20] Mantovani A, Sozzani S, Locati M, Allavena P, Sica A (2002). Macrophage polarization: Tumor-associated macrophages as a paradigm for polarized M2 mononuclear phagocytes. Trends Immunol..

[21] Hwang I, Kim JW, Ylaya K, Chung EJ, Kitano H, Perry C (2020). Tumor-associated macrophage, angiogenesis and lymphangiogenesis markers predict prognosis of non-small cell lung cancer patients. J. Transl. Med..

[22] Mantovani A, Allavena P, Sica A, Balkwill F (2008). Cancer-related inflammation. Nature.

[23] Zhong Q, Fang Y, Lai Q, Wang S, He C, Li A (2020). CPEB3 inhibits epithelial-mesenchymal transition by disrupting the crosstalk between colorectal cancer cells and tumor-associated macrophages via IL-6R/STAT3 signaling. J. Exp. Clin. Cancer Res..

[24] Lopes RL, Borges TJ, Zanin RF, Bonorino C (2016). IL-10 is required for polarization of macrophages to M2-like phenotype by mycobacterial DnaK (heat shock protein 70). Cytokine.

[25] Fre S, Pallavi SK, Huyghe M, Lae M, Janssen KP, Robine S (2009). Notch and Wnt signals cooperatively control cell proliferation and tumorigenesis in the intestine. Proc. Natl. Acad. Sci. U. S. A..

[26] Arcaroli JJ, Tai WM, McWilliams R, Bagby S, Blatchford PJ, Varella-Garcia M (2016). A NOTCH1 gene copy number gain is a prognostic indicator of worse survival and a predictive biomarker to a Notch1 targeting antibody in colorectal cancer. Int. J. Cancer.

[27] Jackstadt R, van Hooff SR, Leach JD, Cortes-Lavaud X, Lohuis JO, Ridgway RA (2019). Epithelial NOTCH signaling rewires the tumor microenvironment of colorectal cancer to drive poor-prognosis subtypes and metastasis. Cancer Cell.

[28] Chanrion M, Kuperstein I, Barrière C, El Marjou F, Cohen D, Vignjevic D (2014). Concomitant Notch activation and p53 deletion trigger epithelial-to-mesenchymal transition and metastasis in mouse gut. Nat. Commun..

[29] Jass JR (2007). Classification of colorectal cancer based on correlation of clinical, morphological and molecular features. Histopathology.

[30] van Denderen BJ, Thompson EW (2013). Cancer: The to and fro of tumour spread. Nature.

[31] Giese A, Loo MA, Tran N, Haskett D, Coons SW, Berens ME (1996). Dichotomy of astrocytoma migration and proliferation. Int. J. Cancer.

[32] Fedotov S, Iomin A (2007). Migration and proliferation dichotomy in tumor-cell invasion. Phys Rev Lett..

[33] Murray PJ (2017). Macrophage polarization. Annu. Rev. Physiol..

[34] Fridman WH, Pagès F, Sautès-Fridman C, Galon J (2012). The immune contexture in human tumours: Impact on clinical outcome. Nat. Rev. Cancer.

[35] Zhang Y, Sime W, Juhas M, Sjölander A (2013). Crosstalk between colon cancer cells and macrophages via inflammatory mediators and CD47 promotes tumour cell migration. Eur. J. Cancer.

[36] Pinto ML, Rios E, Durães C, Ribeiro R, Machado JC, Mantovani A (2019). The two faces of tumor-associated macrophages and their clinical significance in colorectal cancer. Front. Immunol..

[37] Ibrahim ML, Klement JD, Lu C, Redd PS, Xiao W, Yang D (2018). Myeloid-derived suppressor cells produce IL-10 to elicit DNMT3b-dependent IRF8 silencing to promote colitis-associated colon tumorigenesis. Cell Rep..

[38] Waldner MJ, Neurath MF (2014). Master regulator of intestinal disease: IL-6 in chronic inflammation and cancer development. Semin. Immunol..

[39] Chen L, Wang S, Wang Y, Zhang W, Ma K, Hu C (2018). IL-6 influences the polarization of macrophages and the formation and growth of colorectal tumor. Oncotarget.

[40] He G, Dhar D, Nakagawa H, Font-Burgada J, Ogata H, Jiang Y (2013). Identification of liver cancer progenitors whose malignant progression depends on autocrine IL-6 signaling. Cell.

[41] Lièvre A, Bachet JB, Le Corre D, Boige V, Landi B, Emile JF (2006). KRAS mutation status is predictive of response to cetuximab therapy in colorectal cancer. Cancer Res..

[42] Douillard J-Y, Oliner KS, Siena S, Tabernero J, Burkes R, Barugel M (2013). Panitumumab-FOLFOX4 treatment and RAS mutations in colorectal cancer. N. Engl. J. Med..

[43] Ostrem JM, Peters U, Sos ML, Wells JA, Shokat KM (2013). K-Ras(G12C) inhibitors allosterically control GTP affinity and effector interactions. Nature.

[44] Chen DS, Mellman I (2017). Elements of cancer immunity and the cancer-immune set point. Nature.

[45] André T, Shiu KK, Kim TW, Jensen BV, Jensen LH, Punt C (2020). Pembrolizumab in microsatellite-instability-high advanced colorectal cancer. N. Engl. J. Med..

