# Supplementary material for: Loss of HES1 expression is associated with extracellular matrix remodeling and tumor immune suppression in KRAS mutant colon adenocarcinomas
Source: Sci Rep. 2023 Sep 25;13:15999. doi: 10.1038/s41598-023-42234-7 (PMC10519992; doi:10.1038/s41598-023-42234-7)
Supplement: Supplementary file 1 — Supplementary Information. [file 41598_2023_42234_MOESM1_ESM.docx]

**Supplemental Data**

**
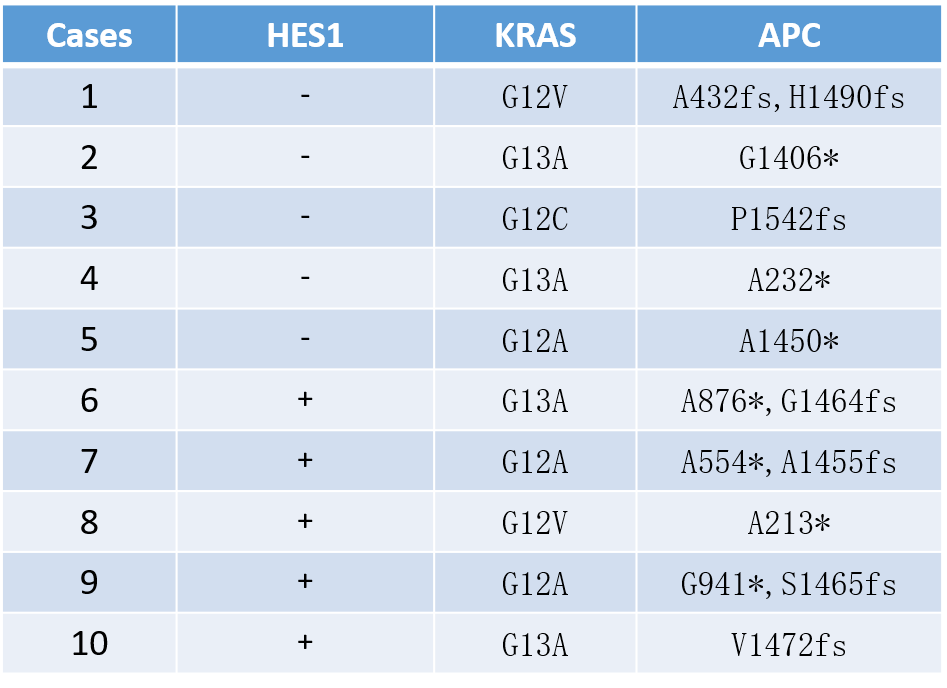
**

fs: Frameshift mutation; *: Nonsense mutation.

**Table S1. Mutation Types of KRAS and APC of cohort 1**

**
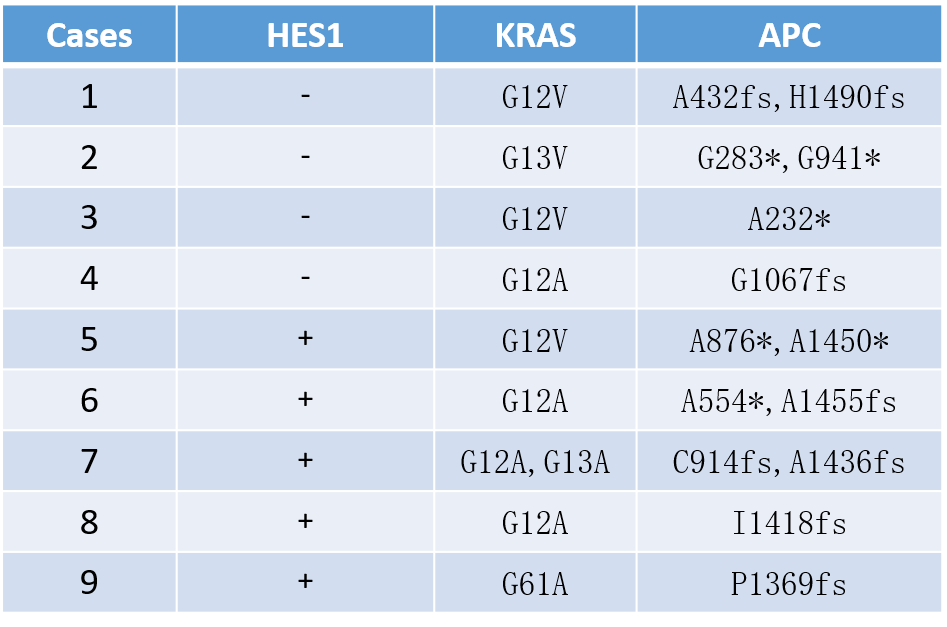
**

fs: Frameshift mutation; *: Nonsense mutation.

**Table S2. Mutation types of KRAS and APC of cohort2**

**Supplemental Figure Legends**

**Fig S1. HES1 expression pattern in CRC.** Representative HES1 immunohistochemistry staining shows HES1 positive reactivity with strong nuclear expression pattern in CRC tumor cells, referred as HES1 (+) (A), and loss of HES1 expression in CRC tumor cells, referred as HES1 (-) (B). HES1 was expressed in the nuclei of stromal cells and immune cells in both groups.

**Fig S2.** **Nanostring RNA gene expression array analyses** **of *KRAS* WT CRCs.** (A) Volcano plot identified 12 DEGs between HES1 (+) group (n=5) and HES1 (-) group (n=5) in KRAS WT CRCs (p<0.05, abs (logFC)>1), of which 6 DEGs were upregulated and 6 DEGs were downregulated in HES1 (-) group. (B-C) Heatmaps of matrix remodeling and metastasis process-related genes (B) and cell proliferation process-related genes (C) were shown for 12 KRAS WT CRCs including HES1 (-) (blue bar) and HES1 (+) cases (red bar). (D) The GSVA plot showing that there was no significant correlation with HES1-loss.

**Fig S3 (related to Fig 4 and Fig 6; original membranes were shown below each panel). HES1 downregulation and the impact on IL10 expression in CRC cells.** (A) HES1 expression in CRC cell lines and HES1 knockdown by shRNA (A: SW480; B: RKO; C: LS174T; D: HT29; E: HCT116; F: SW620). Among these, HT29 has WT KRAS; RKO and HCT116 are MSI cells; LS174T and SW480 were not included in Fig 4 & 6 because of technical challenge in cell migration analysis. (B) IL10 expression increased in LS174T and SW620 cells with HES1 downregulation. Blots shown were representative of three similar experiments.

**Fig S4. Mutation profiles of HES1-high and HES-low CRC in TCGA.**

**Supplemental Figures**

**Fig S1**

**
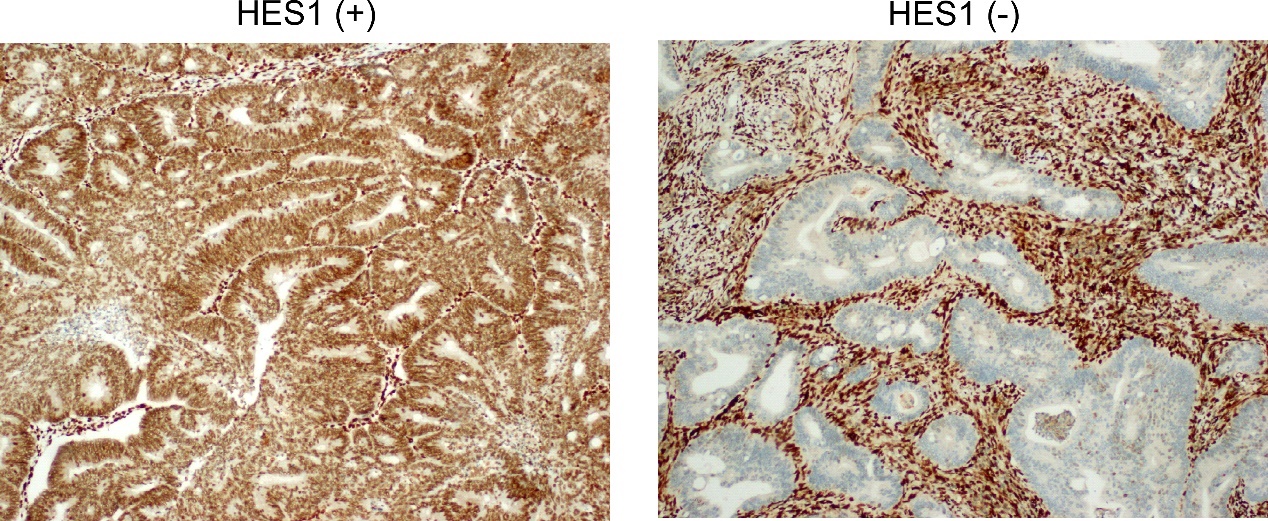
**

**Fig S2**

**
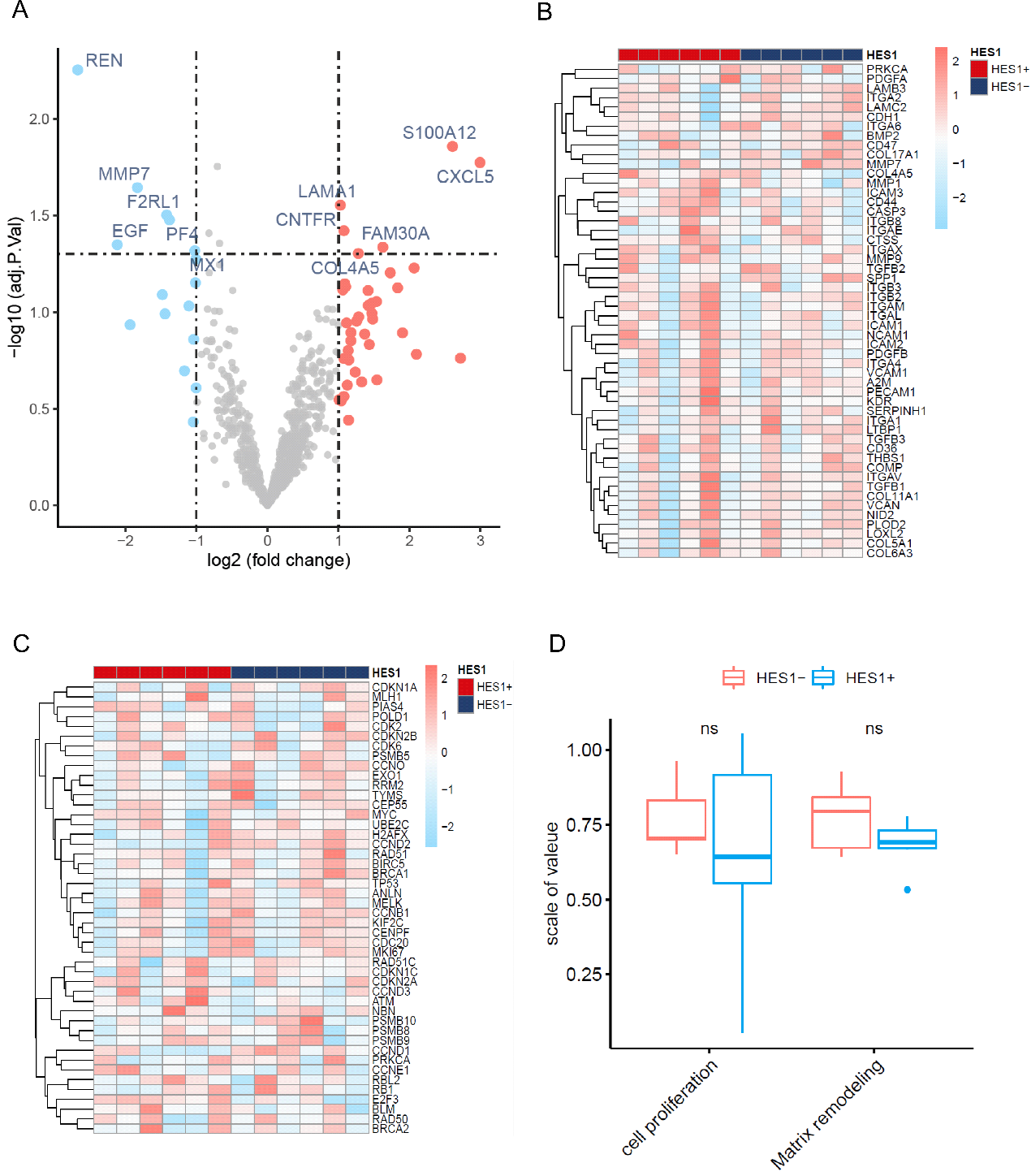
**

**Fig S3**

**
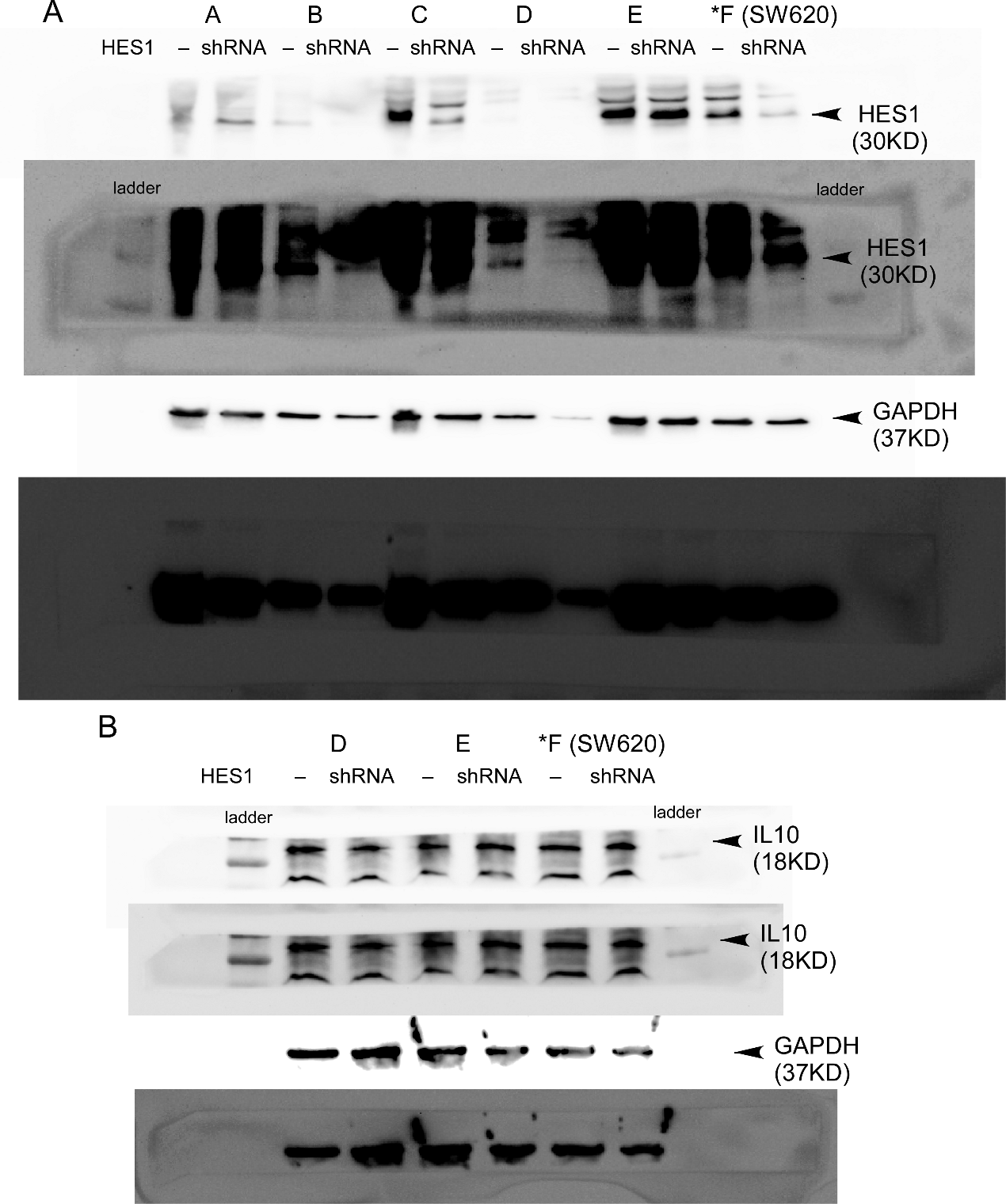
**

**Fig S4**

**
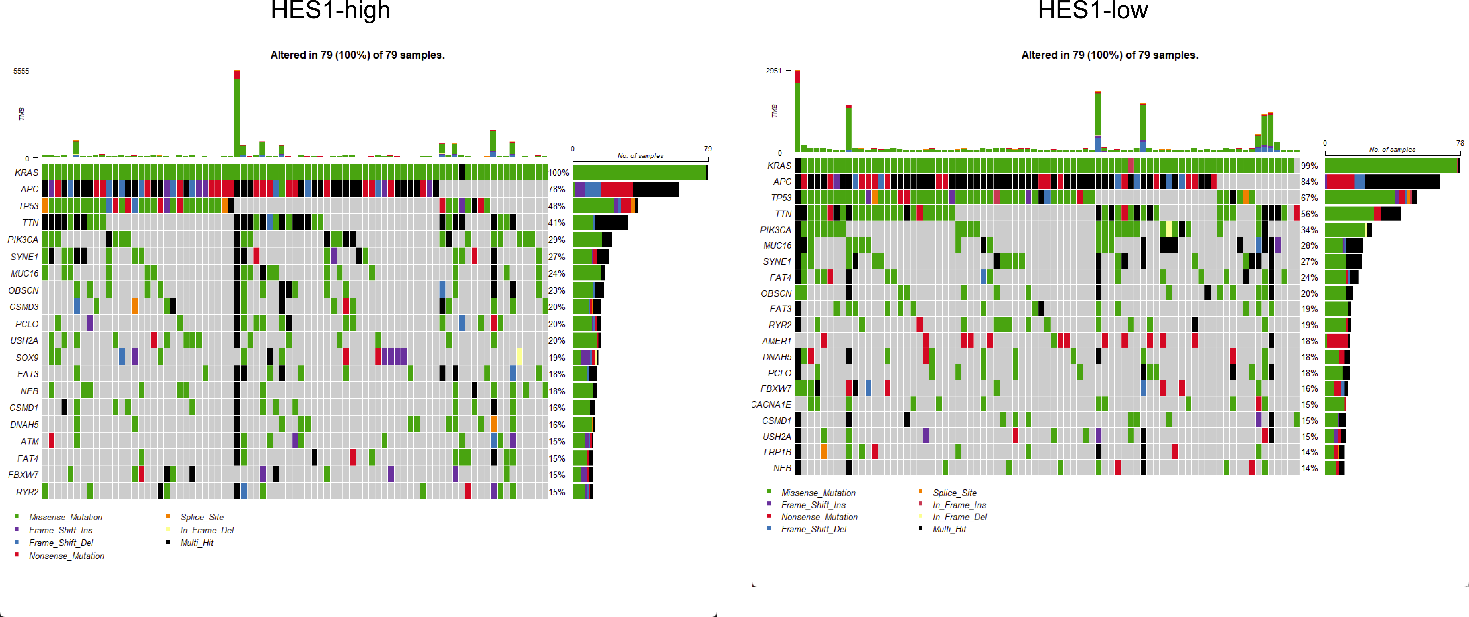
**
